# Exogenous hydrogen sulfide alleviates cerebral ischemia/reperfusion injury via inhibiting RIPK1-mediated neuronal necroptosis and ROS overaccumulation

**DOI:** 10.3389/fmolb.2026.1968646

**Published:** 2026-10-14

**Authors:** Chuanjiang Huang, Hao Shang, Zhihao Fang, Lei Zhu, Chao Chen, Yanan Zang

**Affiliations:** 1 Department of Neurosurgery, The First Affiliated Hospital of Shandong First Medical University and Shandong Provincial Qianfoshan Hospital, Jinan, China; 2 Department of Neurosurgery, Jiangxi Provincial People’s Hospital, The First Affiliated Hospital of Nanchang Medical College, Nanchang, China; 3 Nanchang University, Nanchang, China; 4 Department of Neurosurgery, The Second Affiliated Hospital of Shandong First Medical University, Taian, China; 5 Department of Cadre Ward, The 960th Hospital of the PLA Joint Logistics Support Force, Jinan, China

**Keywords:** cerebral I/R injury, hydrogen sulfide, necroptosis, ripk1, ROS

## Abstract

**Background:**

Cerebral I/R injury is a significant pathological factor contributing to neuronal death and neurological dysfunction following stroke. Necroptosis and excessive oxidative stress are pivotal mechanisms that drive ischemic neuronal damage. H_2_S has been identified as a neuroprotective gaseous signaling molecule; however, its regulatory role in neuronal necroptosis during cerebral I/R injury remains inadequately understood.

**Methods:**

*In vitro*, we established a cell OGD model to simulate neuronal I/R injury. Following pretreatment with NaHS or Necrostatin-1, we assessed cell viability, the necroptotic rate, intracellular ROS levels, mitochondrial membrane potential, and the expression of necroptosis-related proteins. *In vivo*, we established a mouse model of MCAO/R. After NaHS and Nec-1 intervention, we evaluated neurological deficit scores, cerebral blood perfusion, brain water content, cerebral infarct volume, neuronal ultrastructure, and the expression of RIPKs proteins.

**Results:**

OGD treatment resulted in a time-dependent decline in SH-SY5Y cell viability, a significant increase in the rate of neuronal necroptosis, excessive accumulation of ROS, mitochondrial dysfunction, and elevated expression levels of RIPKs and MLKL. Pretreatment with moderate doses of NaHS significantly enhanced cell viability, mitigated ROS overproduction, restored mitochondrial membrane potential, and inhibited OGD-induced neuronal necroptosis. Both NaHS and Nec-1 effectively suppressed the activation of the RIPK1 mediated signaling pathway. *In vivo*, MCAO/R induced severe neurological dysfunction, cerebral hypoperfusion, brain edema, extensive cerebral infarction, and neuronal ultrastructural damage. NaHS or Nec-1 treatment significantly improved neurological deficits, restored regional cerebral blood flow, reduced brain edema and infarct volume, and alleviated neuronal morphological damage by downregulating the expression of RIPK1 and RIPK3.

**Conclusion:**

Necroptosis played a crucial role in acute cerebral I/R injury, both *in vitro* and *in vivo*, mediated by RIPK1. Exogenous H_2_S or Nec-1 exhibited significant neuroprotective effects against cerebral I/R injury by suppressing RIPK1-mediated neuronal necroptosis and inhibiting ROS. These findings provided a novel theoretical basis for the clinical management of ischemic stroke.

## 1 Introduction

Ischemia stroke results in significant morbidity, disability, and mortality globally, with ischemic events constituting 80%–85% of all stroke cases (Collaborators, 2019). Intravenous thrombolysis and mechanical thrombectomy represent the most effective treatments for acute cerebral infarction, as they can salvage the penumbra, dissolve the thrombus, and rapidly restore blood reperfusion (Powers, 2020). However, following the restoration of cerebral blood supply after prolonged ischemia, local inflammation and excessive reactive oxygen species (ROS) can induce secondary brain injury and neuronal cell death (Datta et al., 2020), leading to cerebral ischemia–reperfusion injury (CIRI). CIRI is critical in the onset and progression of cerebral ischemic diseases (Carbone et al., 2019). The mechanisms underlying neural cell death in CIRI have become a focal point for medical researchers, encompassing apoptosis, necrosis, necroptosis, and autophagy.

Necroptosis, also known as programmed necrosis, is initiated by death receptor agonists such as TNF-α, FasL, and TRAIL. This regulated form of necrotic cell death is recognized as one of the several cell death modalities observed post-cerebral ischemia and ischemia/reperfusion (I/R) injury. It represents a caspase-independent mechanism of cell demise, induced by energy depletion, oxidative stress, and reactive oxygen species (ROS) accumulation. The morphological hallmarks of necroptosis involve organelle swelling and plasma membrane rupture, contingent upon necrosome assembly. This complex comprises receptor-interacting protein kinase-1 (RIPK1), receptor-interacting protein kinase-3 (RIPK3), and mixed lineage kinase domain-like protein (MLKL) (Huang et al., 2013; de Almagro and Vucic, 2015). The patterns of cell death in CIRI are complex; however, ongoing research indicates that the type of cell death correlates with ischemia duration, the degree of aerobic metabolism, and residual blood flow. Moderate CIRI may induce autophagy and activate survival recovery systems, whereas severe injury primarily triggers apoptotic and necrotic pathways (Zhang et al., 2022). Amani et al. report that oxidative stress may induce necroptosis in the CA1 region of the hippocampus (Amani et al., 2019). Increasing evidence suggests that RIPK1 and RIPK3 interact with MLKL through its kinase domain, leading to the phosphorylation of MLKL. This process activates necroptosis and contributes to cellular damage, highlighting the significant mediating roles of these kinases in necroptosis (Choi et al., 2019; Robinson et al., 2019). Necrostatin-1 (Nec-1) protects cells from necroptotic death by reducing RIPK1 kinase activity through the inhibition of RIPK1 serine phosphorylation at multiple residues (Ofengeim and Yuan, 2013). Nonetheless, the upstream regulatory factors and targeted intervention strategies for neuronal necroptosis in oxygen-glucose deprivation (OGD)-induced neuronal injury and middle cerebral artery occlusion/reperfusion (MCAO/R) brain injury require further investigation.

Hydrogen sulfide (H_2_S), an endogenous gaseous signaling molecule, plays a crucial role in regulating various physiological and pathological processes within the central nervous system, including neuroprotection, antioxidation, anti-inflammation, and anti-apoptosis (Huang et al., 2023). Sodium hydrosulfide (NaHS, an exogenous H_2_S donor), a stable exogenous H_2_S donor, can continuously release H_2_S both *in vivo* and *in vitro*. Previous studies have demonstrated that H_2_S can mitigate cerebral I/R injury; however, the extent to which its neuroprotective effects are linked to the regulation of neuronal necroptosis remains inadequately explored. Notably, there is a scarcity of systematic biochemical and morphological evidence to elucidate the influence of H_2_S on the necroptotic signaling pathway during neuronal I/R injury. Consequently, elucidating the role of H_2_S in the cerebral ischemia/reperfusion injury process is of significant importance for the prevention and treatment of I/R injury. This study, therefore, aims to investigate whether and to what extent exogenous hydrogen sulfide can alleviate acute ischemia/reperfusion injury by inhibiting cellular necroptosis.

Pharmacological analysis has demonstrated that treatment with Nec-1, a necroptosis inhibitor (Degterev et al., 2005), can reduce neuronal death and infarct volume in various animal models, including MCAO (Deng et al., 2019), traumatic brain injury (TBI) (Cho et al., 2011), and hypoxia–ischemia (HI) (Chavez-Valdez et al., 2012). These findings indirectly suggest that necroptosis may play a role in the pathogenesis of neuronal death following different types of brain injury. However, whether mitochondrial dysfunction and oxidative stress in CIRI are associated with necroptosis (Ferrada et al., 2020; Gong et al., 2022), or whether H_2_S influences cell CIRI through the necroptotic pathway, was the focus of the present study. We employed a cellular OGD model and a mouse MCAO model to simulate cerebral ischemia-reperfusion injury and comprehensively examined the status of necroptosis in this context. Additionally, we utilized exogenous injections of NaHS to validate the regulatory role of H_2_S in necroptosis.

In this study, we employed a MCAO model in mice and a cell OGD model, utilized various approaches to explore the potential roles of RIPK-dependent necroptosis in the pathogenesis of acute ischemic brain injury. Our findings indicated that cerebral ischemia elevated the expression levels of RIP1 and RIPK3 in the brain. Furthermore, H_2_S effectively mitigated the ischemia-induced alterations mentioned above while also protecting the brain from ischemic injury and functional deficits. These data provide new experimental evidence supporting the clinical application of H_2_S in the treatment of ischemic stroke and suggest that ROS are involved in RIPK-dependent neuronal necroptosis following cerebral ischemia. This study also offers novel insights into the pathological mechanisms underlying neuronal damage in the brain after ischemic stroke.

## 2 Materials and methods

### 2.1 Reagents and antibodies

Anti-RIPK1, anti-RIPK3, and anti-MLKL antibodies were obtained from Abcam (Cambridge, MA, USA). DMEM medium was sourced from Gibco (Rockville, MD, USA). Fetal bovine serum (FBS) was procured from Life Technologies (Grand Island, NY, USA). The protein concentration assay kit was acquired from Biorad Laboratory (Hercules, CA, USA). ECL Western blotting detection reagents were supplied by Amersham Company (Piscataway, NJ, USA). PVDF membranes were obtained from Millipore Company (Billerica, MA, USA). Additional reagents were sourced from Sigma Company (St. Louis, MO, USA).

### 2.2 Cell line and OGD modeling

SH-SY5Y cells were procured from the Shanghai Institute of Cell Biology, Chinese Academy of Sciences (Shanghai, China). The cells were cultured in DMEM medium supplemented with 10% fetal bovine serum, 2 mmol/L glutamine (Gibco, Grand Island, NY, USA), penicillin (100 U/mL), and streptomycin (100 mg/mL), and were maintained at 37 °C in a humidified atmosphere containing 5% CO_2_. To establish models of OGD/R induced cell injury, SH-SY5Y cells were washed three times with pre-warmed PBS and subsequently placed in serum- and glucose-free medium. The cells were then incubated in an oxygen-free environment (95% N_2_, 5% CO_2_) within an automated incubator (BioTek, Winooski, VT, United States) for durations ranging from 2 to 12 h, After OGD treatment, the serum- and glucose-free medium was replaced with normal complete culture medium, and cells were cultured under normoxic conditions (37 °C, 5% CO_2_) for 24 h reoxygenation to simulate reperfusion. Cells in the control group were cultured continuously in normal high-glucose medium under normoxic conditions without OGD/R exposure (Luo et al., 2017).

### 2.3 Cell viability assay

SH-SY5Y cells were plated in a 96-well microplate and incubated for 24 h. Subsequently, the cells were treated with NaHS for 2 h before subjecting them to OGD incubation. MTT solution at a concentration of 0.5 mg/mL in PBS was then added to each well to assess cell viability. The formazan crystals were solubilized using 150 µL of DMSO, and the absorbance was measured at 570 nm using an automated multi-well spectrophotometer (Bio-Rad, Richmond, CA, USA), following previously established protocols (Huang et al., 2013).

### 2.4 Measurement of intracellular ROS levels

The average level of intracellular ROS in SH-SY5Y cells was assessed using the redox-sensitive dye DCFH-DA (Molecular Probes, Eugene, OR). The SH-SY5Y cells were incubated in a glucose- and fetal bovine serum (FBS)-free medium under an oxygen-free environment (37 °C, 95% N_2_, 5% CO_2_) for varying durations. Additionally, cells were pretreated with 100 mmol/L Nec-1, 400 mmol/L NAC, or different concentrations of hydrogen sulfide, with cells treated with phosphate-buffered saline (PBS) serving as the control. Following these treatments, the cells were cultured in conventional medium for 24 h. All experimental cells underwent two washes with PBS and were stained in the dark for 30 min with 20 mmol/L DCFH-DA before harvesting. The cells were then lysed with 1% Triton X-100, and fluorescence was measured at an excitation wavelength of 485 nm and an emission wavelength of 530 nm using a fluorescence spectrometer (HTS 7000, Perkin Elmer, Boston, MA).

### 2.5 Flow cytometry analysis of programmed cell death and measurement of mitochondrial membrane potential

SH-SY5Y cells, subjected to various treatments, were harvested at specified post-treatment time points. Cell death was assessed using the annexin V-FITC necroptosis detection kit (Invitrogen, Grand Island, NY) following the manufacturer’s protocol. Initially, SH-SY5Y cells were harvested, washed with PBS twice, and then resuspended in 400 μL of 1× binding buffer (10 mM HEPES/NaOH, 140 mM NaCl, 2.5 mM CaCl2, pH 7.4). Subsequently, 100 μL of cells were combined with 5 μL of annexin V-FITC and 10.0 μL of propidium iodide in a 5-mL culture tube, followed by a 15-min incubation at room temperature in the dark. Upon addition of 1× binding buffer to each tube, flow cytometry analysis (FACScan, Becton Dickinson, San Jose, CA) was performed on the stained cells. Cell death rates were determined using CELL quest software (Becton Dickinson), with data acquisition involving 20,000 cells per tube, and quantification of viable and dead cells under each experimental condition.

Mitochondrial membrane potential was assessed based on Rhodamine 123 dye retention (Beyotime Biotech, Nanjing, China). The harvested cells were washed with PBS twice, exposed to 10.0 μg/mL Rhodamine 123 at 37 °C for 30 min, washed again with PBS, and the fluorescence intensities of Rhodamine 123 were measured using flow cytometry.

### 2.6 Animals

Adult male wildtype C57/BL6J mice, aged 6–8 weeks, were obtained from Jinan Pengyue Animal Co., Ltd. The animal experimental procedures received approval from the Animal Care Committee of Shandong Provincial Qianfoshan Hospital (Date: 3 December 2021; Approval number: S1175). The mice were housed in the animal facility of Shandong Provincial Qianfoshan Hospital under a 12 h light/12 h dark cycle, with unrestricted access to standard food and water for 1 week prior to the experiments.

### 2.7 Establishment of mice MCAO models and lateral ventricle injection

To investigate cell death patterns in CIRI *in vivo*, an experimental MCAO model was established in mice. Transient focal cerebral ischemia was induced by MCAO following intraperitoneal anesthesia with pentobarbital sodium. An incision was made in the neck’s midline to gently dissect the right carotid artery and vagus nerve. Temporary ligation of the right common carotid and internal carotid arteries was performed using an arteriole clip. Subsequently, after ligating the external carotid artery, a small incision was made to insert a poly-l-lysine-coated monofilament through the internal carotid artery into the middle cerebral artery. Reperfusion was initiated by withdrawing the nylon monofilament after 2 h of blockage. Sham-operated control mice underwent an identical procedure without filament induction. Mice’s body temperature was maintained at 37.5 °C and monitored during the procedure. Relative rCBF was continuously monitored using laser Doppler measurement (PeriFlux System 5,000) throughout the surgery. A reduction of at least 70% from the initial LDF reading was considered indicative of successful occlusion of the MCA perfusion territory (Dinapoli et al., 2006). Unsuccessful candidates were excluded, and the sample size was adjusted by creating qualified models using mice from the same batch.

### 2.8 Experimental design and animal groups

The mice were randomly assigned to four groups: (1) the sham group, (2) the MCAO group, (3) the MCAO + NaHS group, and (4) the MCAO + NEC group, with n = 3 animals per group. Nec-1 and NAC were administered into the lateral ventricle of the mice using intracerebroventricular (ICV) injection at stereotaxic coordinates relative to bregma: AP −0.5 mm, ML +1.5 mm, DV −2.2 mm. A total volume of 1 μL solution was infused at a rate of 0.2 μL/min, with a 5-min dwell time before slow needle withdrawal. Nec-1 treatment involved dissolving Nec-1 in anhydrous DMSO to a concentration of 2.6 μg/μL, with 1.0 μL solution (2.6 μg Nec-1 per mouse) injected ICV. For NAC treatment, NAC was prepared in sterile PBS (pH 7.4) at 100.0 mM, and 1.0 μL was administered intracerebroventricularly, corresponding to a total dose of 100.0 nmol (16.3 μg) NAC per animal. The sham group and MCAO group animals received injections of the same volume of sterile PBS at the same time points.

### 2.9 Neurologic deficit scores

Neurological function was evaluated with the modified Bederson 0–5 scoring system and Zea-Longa 0–4 scoring system at 1, 3, and 6 days post-modeling under blinded assessment (Bederson et al., 1986). Zea-Longa grading: 0 = intact; 1 = contralateral forelimb flexion on tail lift; 2 = spontaneous contralateral circling; 3 = contralateral falling tendency; 4 = no spontaneous movement plus lethargy. Modified Bederson grading: 0 = normal; 1 = incomplete contralateral forelimb extension; 2 = reduced lateral resistance to paretic side push; 3 = sustained contralateral circling; 4 = recurrent contralateral falling; 5 = complete loss of spontaneous movement or stupor.

### 2.10 TTC staining and infarct volume measurement

Mice were placed in an anesthesia induction chamber. Anesthesia was induced with 5% isoflurane. Afterwards, the concentration was maintained at 2% isoflurane to monitor vital signs. While the animals remained under deep anesthesia and before any signs of respiratory or cardiac arrest occurred, cervical dislocation was performed as the primary euthanasia method to irreversibly disrupt the central nervous system and ensure painless death.

Following euthanasia of the animals using 2% isoflurane, the whole brain was extracted intact on ice, promptly frozen at −20 °C for 20 min, and subsequently sectioned into six slices, each approximately 2 mm thick. These brain sections were then submerged in a 2.0% (wt/vol) solution of TTC and subjected to a 30-min incubation at 37 °C in darkness. Normal brain tissue exhibited a red stain, while the infarcted brain tissue appeared white. After fixation in 4% formalin for 24 h, the volume of infarction in the cerebral hemisphere was quantified and assessed using ImageJ software (Zhang et al., 2014).

### 2.11 Transmission electron microscopy

A brain mass measuring approximately 1 × 1 × 3 mm^3^ was extracted from the core region of the infarcted cortex and subsequently fixed in 2.5% glutaraldehyde followed by 1% osmium tetroxide. After dehydration through a series of acetone concentrations, the samples were embedded in a mixture of acetone and embedding medium, then solidified in an oven. Sections of 50–100 nm were obtained using an ultra-thin slicer and were double-stained with 3% uranyl acetate and lead nitrate. Finally, the samples were examined and images were captured using a Hitachi HT7700 transmission electron microscope.

### 2.12 Western blot

Cells or brain tissues were lysed in RIPA buffer (Beyotime Biotechnology) for 30 min on ice, and protein concentration was determined using a BCA protein assay kit (Beyotime Biotechnology). Following a 5-min boiling step at 100 °C, proteins (50 µg) were separated by 10% or 12% SDS–PAGE and subsequently transferred onto PVDF membranes, as previously described (Huang et al., 2013). After blocking with 5% non-fat milk, the membranes were incubated with primary antibodies overnight at 4 °C. β-Actin (1:1,000) served as a loading control. After a 1-h incubation with the appropriate secondary antibodies at room temperature, protein bands were visualized and analyzed using a chemiluminescent imaging system (FluorChem, ProteinSimple, San Jose, CA, United States).

### 2.13 Statistical analysis

All experiments were conducted 3 to 6 times, with each group comprising at least three mice. The quantitative data confirmed normality and homoscedasticity prior to analysis, and results are presented as mean ± standard deviation. Statistical analysis was conducted using analysis of variance (ANOVA). Correlation analysis employed the Pearson product-moment correlation coefficient. All statistical analyses were performed using GraphPad Prism 8 software (GraphPad Software, Inc., La Jolla, CA, USA), with a significance threshold set at p < 0.05. (*p < 0.05, **p < 0.01, ***p < 0.001, and ****p < 0.0001).

## 3 Results

### 3.1 OGD induced cell death which could be rescued by exogenous H_2_S in SH-SY5Y cells

We established an *in vitro* cell model of I/R injury to assess the presence of necroptosis. To create OGD cell models, SH-SY5Y cells were cultured in a glucose- and FBS-free medium under oxygen-deprived conditions (37 °C, 95% N_2_, 5% CO_2_) for varying durations. Following OGD induction, cellular viability was evaluated using the MTT assay. The results depicted in Figure 1A reveal a decline in SH-SY5Y cell viability to 78.6%, 57.6%, 39.3%, and 29.0% after OGD durations of 2.0, 4.0, 6.0, and 8.0 h, respectively. Significant decreases in cellular viability were observed at 4 h post-OGD exposure (57.60% ± 4.85%, P < 0.01). Unlike cells cultured in a standard medium under normoxic conditions (37 °C, 95% air, 5% CO_2_), OGD led to a time-dependent reduction in cell viability. Therefore, a 4-h OGD duration was selected for subsequent investigations.

**FIGURE 1:**
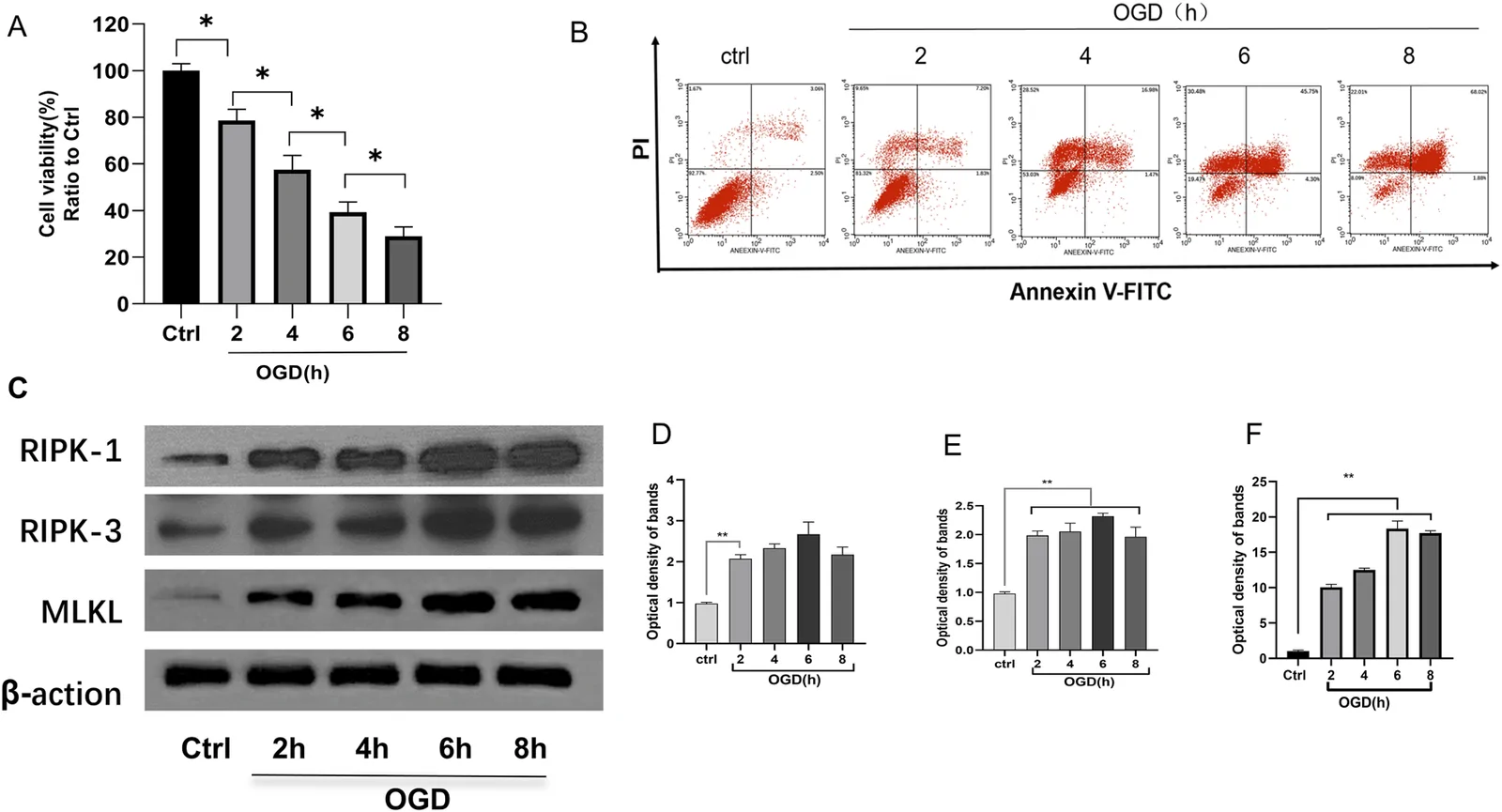
Short-term effects of OGD-induced cell death. **(A)** MTT assay demonstrated that the viability of cells decreased as the duration of OGD increased. **(B)** Flow cytometry analysis indicated that cell death intensified with prolonged OGD exposure. **(C)** Western blot revealed a significant increase in the expression of RIPK1, RIPK3, and MLKL proteins as the duration of OGD extended. **(D,E)** and **(F)** represent the relative expression levels of RIPK1, RIPK3, and MLKL respectively.

To elucidate the neuroprotective impact of NaHS on OGD-induced cell death, SH-SY5Y cells were treated with varying concentrations of NaHS for 1 h, then subjected to OGD for 4 h followed by standard culture conditions for 24 h. Figure 2A illustrates that at NaHS concentrations of 10.0, 30.0, 60.0, 90.0, and 120.0 mmol/L, cell viability after 4 h of OGD increased to 52.3%, 62.1%, 79.2%, 76.9%, and 73.9%, respectively, compared to the control group’s viability of 48.1%. These findings suggest that 60.0 mmol/L NaHS effectively shields SH-SY5Y cells from OGD-induced damage, thus this concentration was selected for subsequent investigations. Moreover, in Figure 3A, extending OGD exposure from 2.0 to 24.0 h reduced SH-SY5Y cell survival from 74.8% to 15.3%. Conversely, pretreatment with 60.0 mmol/L hydrogen sulfide increased cell survival rates to 83.0% and 25.1% after 4, 8, and 12 h, respectively, representing enhancements of 18.2%, 12.1%, and 10.4% in survival rates. These results indicate that pretreatment with hydrogen sulfide at concentrations ranging from 30 to 120 mmol/L for 2–12 h significantly guards against OGD-induced cell death.

**FIGURE 2:**
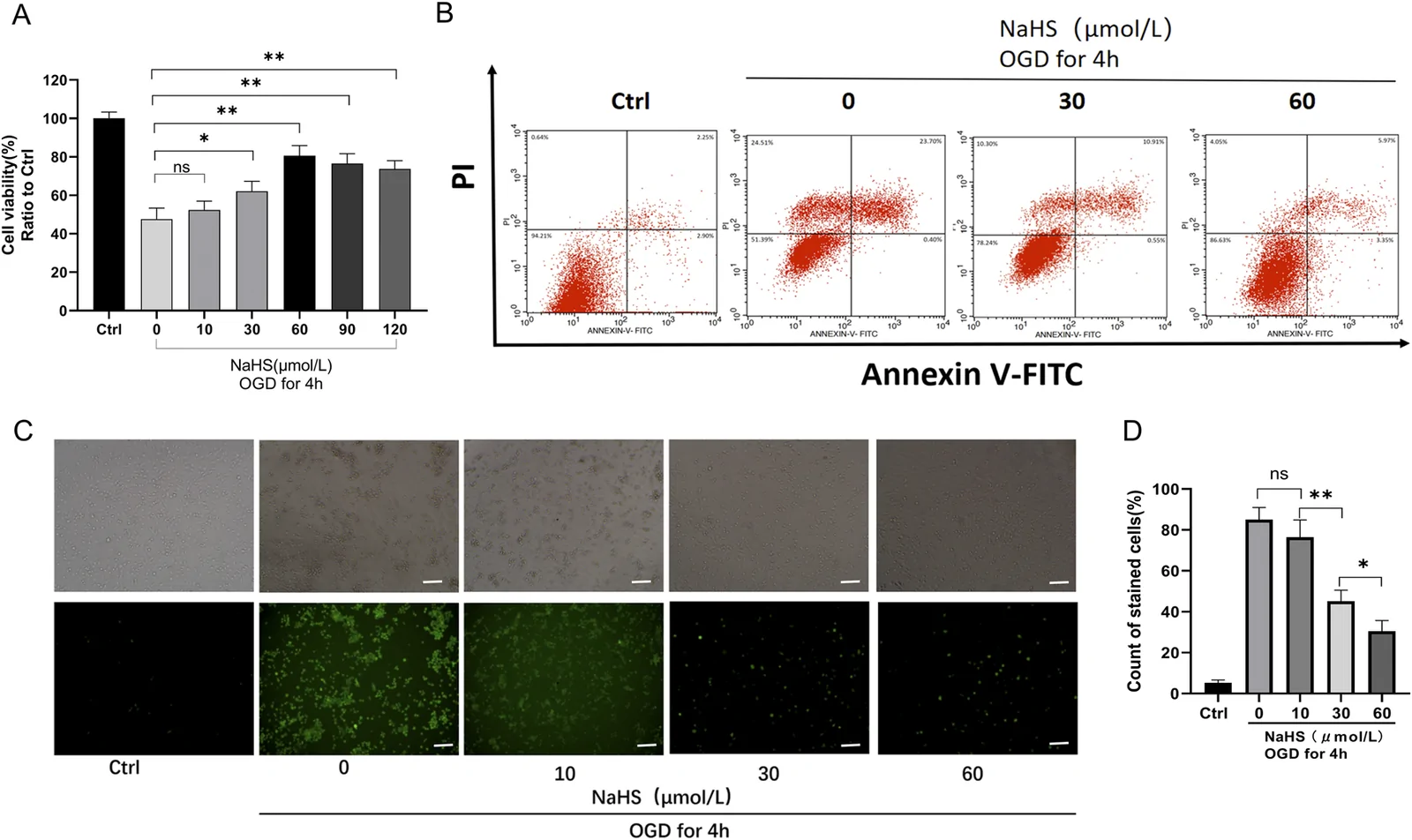
OGD-induced cell necroptosis can be inhibited by exogenous H_2_S and Nec-1. **(A)**MTT assay demonstrated that OGD significantly triggered cell necroptosis, both 60.0 µmol/L NaHS and 100 mmol/L Nec-1 markedly suppressed this process. **(B)**Flow cytometry analysis indicated that both NaHS and Nec-1 suppressed OGD-induced cell necroptosis. **(C)**Western blot revealed cells expressed RIPK1 and RIPK3 significantly increase after 4 h of OGD, both NaHS and Nec-1 suppressed the upregulation of RIPK1 and RIPK3. **(D)**and E represent the relative expression levels of RIPK1 and RIPK3 respectively.

**FIGURE 3:**
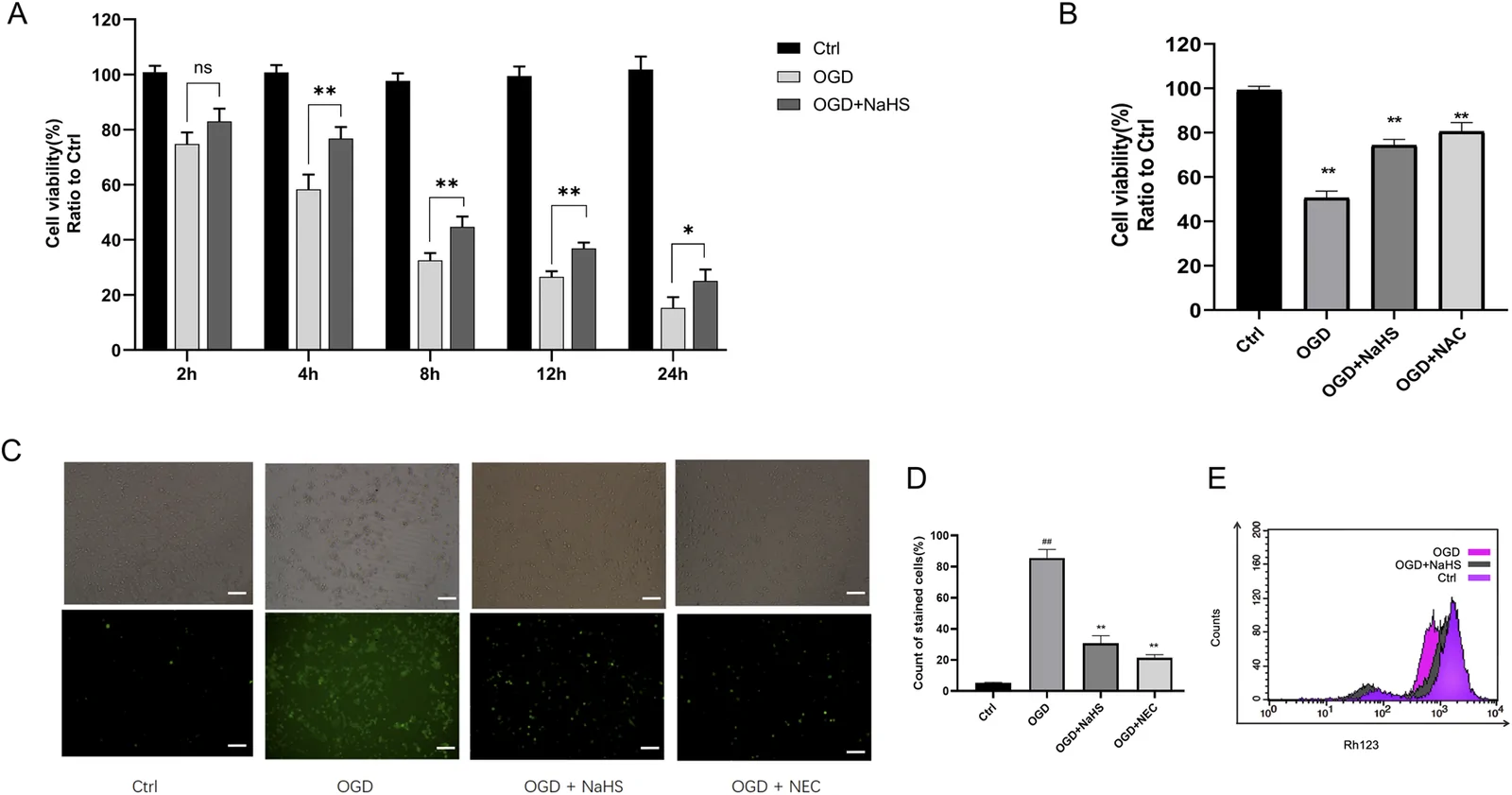
OGD-induced cell death can be inhibited by exogenous H_2_S. **(A)**MTT assay demonstrated that NaHS inhibited OGD-induced cell death in a concentration-dependent manner within a defined range. **(B)**Flow cytometry analysis indicated that NaHS suppressed OGD-induced cell death in a concentration-dependent manner. **(C)**DCFH-DA staining revealed that NaHS inhibited OGD-induced ROS release in a concentration-dependent manner. **(D)**NaHS reduced the proportion of DCFH-DAC-stained cells being incubated by OGD in a concentration-dependent pattern (Flow cytometry). Scale bar = 40 mm.

### 3.2 OGD induced necroptosis which could be rescued by exogenous H_2_S in SH-SY5Y cells

To further investigate the effects and mechanisms of sodium hydrosulfide *in vitro*, an MTT assay was conducted to assess cell viability following a 1-h pretreatment with NaHS or Nec-1. As shown in Figure 4A, after this pretreatment, SH-SY5Y cells were subjected to OGD for 4 h. The viability of SH-SY5Y cells increased from 54.5% to 73.9% and 77.1% following treatment with NaHS and Nec-1, respectively.

**FIGURE 4:**
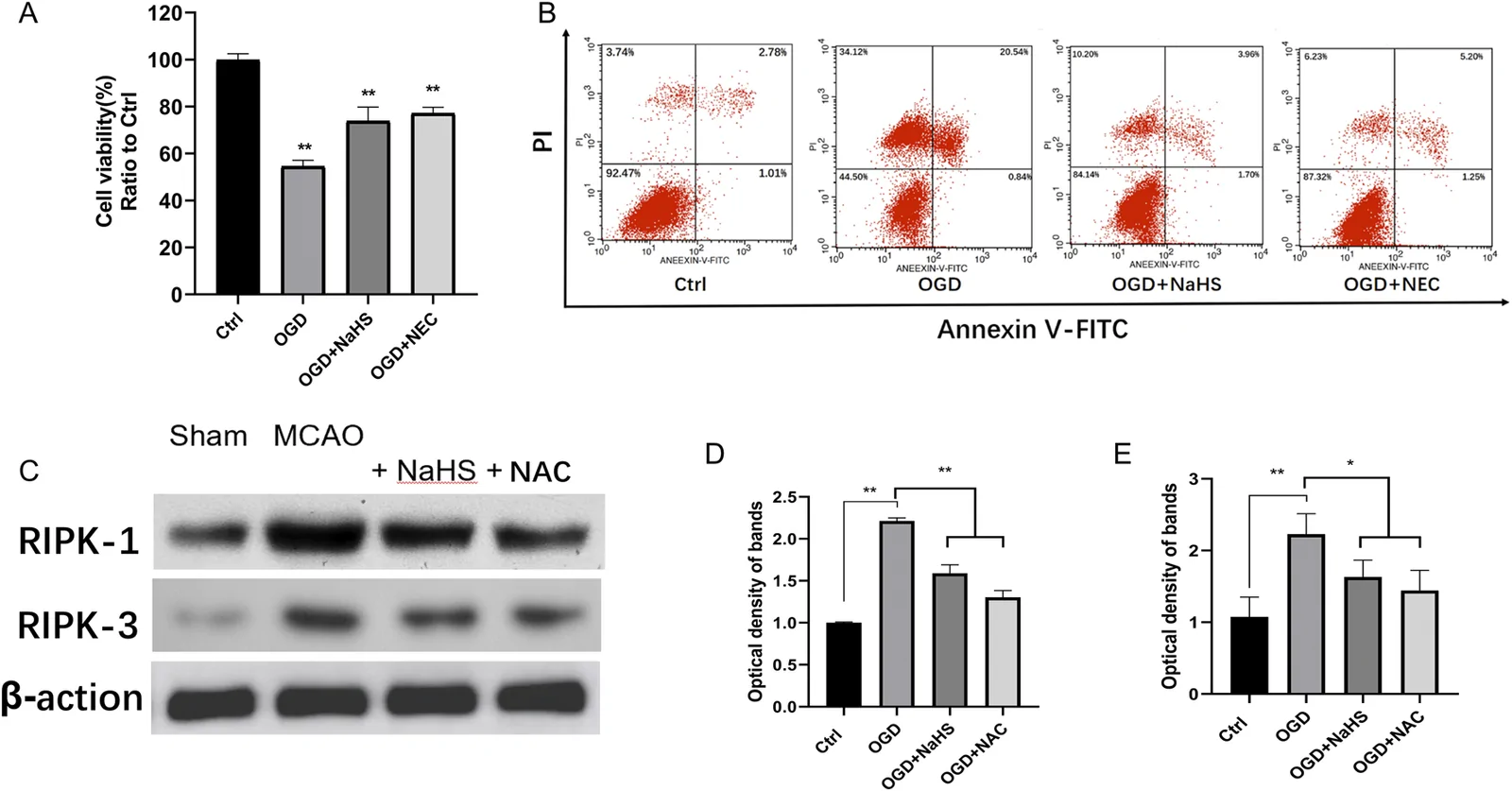
OGD-induced cell death is associated with significant production of ROS. Administration of exogenous H_2_S or Nec-1 can inhibit both cell death and the surge of ROS. **(A)**MTT assay demonstrated that OGD-induced cell death is time-dependent, with hydrogen sulfide capable of reversing this cell death. **(B)**MTT assay demonstrated that OGD significantly triggered cell death, but both 60.0 µmol/L hydrogen sulfide and 400 mmol/L NAC markedly suppressed this process. **(C)**DCFH-DA staining revealed substantial generation of reactive oxygen species (ROS) following reoxygenation of 24 h after 4 h of OGD; both 60.0 µmol/L hydrogen sulfide and 100 mmol/L Nec-1 effectively inhibited cell death and ROS production. **(D)**Hydrogen sulfide and Nec-1 reduced the proportion of DCFH-DAC-stained cells being incubated by OGD (Flow cytometry). **(E)**Evaluation of OGD-induced changes in mitochondrial membrane potential indicated a decrease after 4 h of OGD, which was mitigated by NaHS. Scale bar = 40 mm.

Flow cytometry using Annexin V(AV) and propidium iodide (PI) double staining was conducted to explore the protective mechanisms of sodium hydrosulfide. PI can penetrate the disrupted membrane of necroptotic cells and bind to DNA. Thus, a comprehensive necroptotic response can be elucidated by combining the PI (+)/AV (−) population, indicating cells undergoing necrosis, and the PI (+)/AV (+) population, indicating cells in late necroptosis. However, cells in late apoptosis also exhibit a similar PI (+)/AV (+) staining pattern, indicating that AV/PI staining is not highly specific for distinguishing between apoptosis and necrosis. Nonetheless, a sudden increase in the number of late apoptotic cells without a statistically significant change in the number of early apoptotic cells suggests the involvement of another form of programmed cell death, likely necroptosis. As depicted in Figure 1B, SH-SY5Y cells exposed to OGD for 2h, 4h, 6h, and 8 h before being returned to standard culture conditions for 24 h exhibited necrosis rates of 9.65%, 28.52%, 30.48%, and 22.01%, respectively. Late apoptosis rates were 7.20%, 16.98%, 485.75%, and 68.02%, respectively. However, there were no significant changes in the Annexin V+/PI- ratios. Therefore, based on this experiment, it can be tentatively concluded that cells stained in the (Annexin V-/PI+) and (Annexin V+/PI+) regions correspond to necrotic cells.

As illustrated in Figure 2B, SH-SY5Y cells incubated with NaHS at concentrations of 0 µmol/L, 30.0 µmol/L, and 60.0 µmol/L for 1 h, followed by 4 h of OGD, and subsequently cultured under standard conditions for 24 h, exhibited necrosis (Annexin V-/Pl+) rates of 24.51%, 10.30%, and 4.05%, respectively. The late apoptosis (Annexin V+/Pl+) rates were 23.70%, 10.91%, and 5.97%, respectively. These findings demonstrate that NaHS can protect against OGD-induced cell death, primarily by inhibiting cellular necrosis. Similarly, as shown in Figure 4B, SH-SY5Y cells exposed to OGD for 4 h exhibited necrosis rates of 34.12%. In contrast, pretreatment with NaHS and Nec-1 for 1 h reduced the necrotic cell rates induced by OGD to 10.20% and 6.23%, respectively. This suggests that both NaHS and Nec-1 effectively mitigate OGD-induced cell death in SH-SY5Y cells. Furthermore, the Annexin V+/Pl + rates also decreased from 20.54% to 3.96% with NaHS treatment and to 5.20% with Nec-1 treatment. Thus, these results indicate that exogenous sodium hydrosulfide can partially rescue cells from OGD-induced death by inhibiting necroptosis.

### 3.3 Inhibition of ROS could suppress OGD induced cell necroptosis

Necroptosis is often associated with excessive ROS generation (Ye et al., 2012). To investigate the role of calcium overload and ROS overproduction in I/R induced cell death, we assessed ROS levels using DCFH-DA dye after pre-treating SH-SY5Y cells with the potent ROS scavenger N-acetylcysteine (NAC) at a concentration of 400 mmol/L for 1 h. Cell viability changes were determined by MTT assay, while alterations in cell death patterns were assessed by flow cytometry. Intracellular ROS levels in SH-SY5Y cells were quantified using DCFH-DA dye.

Figure 3B illustrated that treating SH-SY5Y cells with NaHS at a concentration of 60.0 µmol/L or NAC at a concentration of 400 mmol/L for 1 h before subjecting them to OGD for 4 h, followed by conventional culture for 24 h, increased the cell survival rates from 50.65% to 74.30% and 80.57%, respectively. In contrast, Figure 2C demonstrated a significant increase in ROS levels after 4 h of incubation of SH-SY5Y cells compared to the control group, which was effectively mitigated by 10–60 µmol/L NaHS and 100 mmol/L Nec-1 (Figure 3B). Furthermore, MTT assay revealed that OGD-induced cell death in SH-SY5Y cells was notably alleviated by Nec-1 and NAC. Assessment of OGD-induced changes in mitochondrial membrane potential indicated a decrease for 4 h of OGD, which was mitigated by NaHS (Figure 3E). These findings indicate that OGD escalates ROS production in SH-SY5Y cells in a concentration-dependent manner, but pretreatment with H_2_S or Nec-1 significantly reduces ROS production and reverses the decrease in cell viability induced by OGD.

### 3.4 Exogenous H_2_S inhibited necrptosis induced by OGD through suppressing the RIPK1-RIPK3 signaling pathway *in vitro*

To further validate the impact of exogenous NaHS on necroptosis-related protein expression following OGD in SH-SY5Y cells, we utilized western blotting to assess RIPK1, RIPK3, and MLKL levels. Figures 1C–F illustrated that with OGD duration increasing from 2.0 to 8.0 h, RIPK1, RIPK3, and MLKL expression in SH-SY5Y cells notably increased. Conversely, pretreatment with sodium hydrosulfide or Nec-1 suppressed OGD-induced upregulation of RIPK1, RIPK3, and MLKL, indicating that exogenous NaHS and Nec-1 hindered OGD-triggered necroptosis by targeting the RIPK1-RIPK3-MLKL pathway (Figures 4C–E). *In vivo*, exogenous NaHS was observed to attenuate RIPK1, RIPK3, and MLKL protein expression post MCAO/R.

### 3.5 Exogenous H_2_S ameliorated neurological function in mice after MCAO/R injury

MCAO served as a classic model for cerebral ischemia. We examined the therapeutic effects of exogenous H_2_S and Nec-1 on cerebral ischemic injury in a mouse MCAO/R model. Neurological deficit scores were assessed using the Bederson and Zea Longa scales after 1, 3, and 6 days of MCAO/R treatment. At 1 day post-treatment, the Bederson scores in the NaHS group (3.33 ± 0.52) and the NEC group (3.50 ± 0.54) were slightly lower than that of the model group (3.67 ± 0.52). However, by the third day, the Bederson scores in the sodium hydrosulfide group (2.33 ± 0.52) and the NEC group (2.50 ± 0.54) were significantly reduced compared to the model group (3.33 ± 0.52). Furthermore, on the sixth day, the Bederson scores in the sodium hydrosulfide group (1.67 ± 0.62) and the NEC group (1.83 ± 0.41) were significantly lower than those in the model group (2.67 ± 0.46) (P < 0.01, Figures 5A,B). The Zea Longa scores were slightly reduced in the NaHS group (2.43 ± 0.53) and the NEC group (2.46 ± 0.54) compared to the model group (2.86 ± 0.38) on the first day post MCAO/R treatment. However, the Bederson scores in the NaHS group (1.57 ± 0.53) and the NEC group (1.70 ± 0.44) were significantly lower than those in the model group (2.29 ± 0.49) on the third day after MCAO/R treatment. Furthermore, on the sixth day post MCAO/R treatment, the Bederson scores in the NaHS group (1.14 ± 0.38) and the NEC group (1.29 ± 0.49) were significantly lower compared to those in the model group (2.00 ± 0.58) (P < 0.01, Figures 5A,C).

**FIGURE 5:**
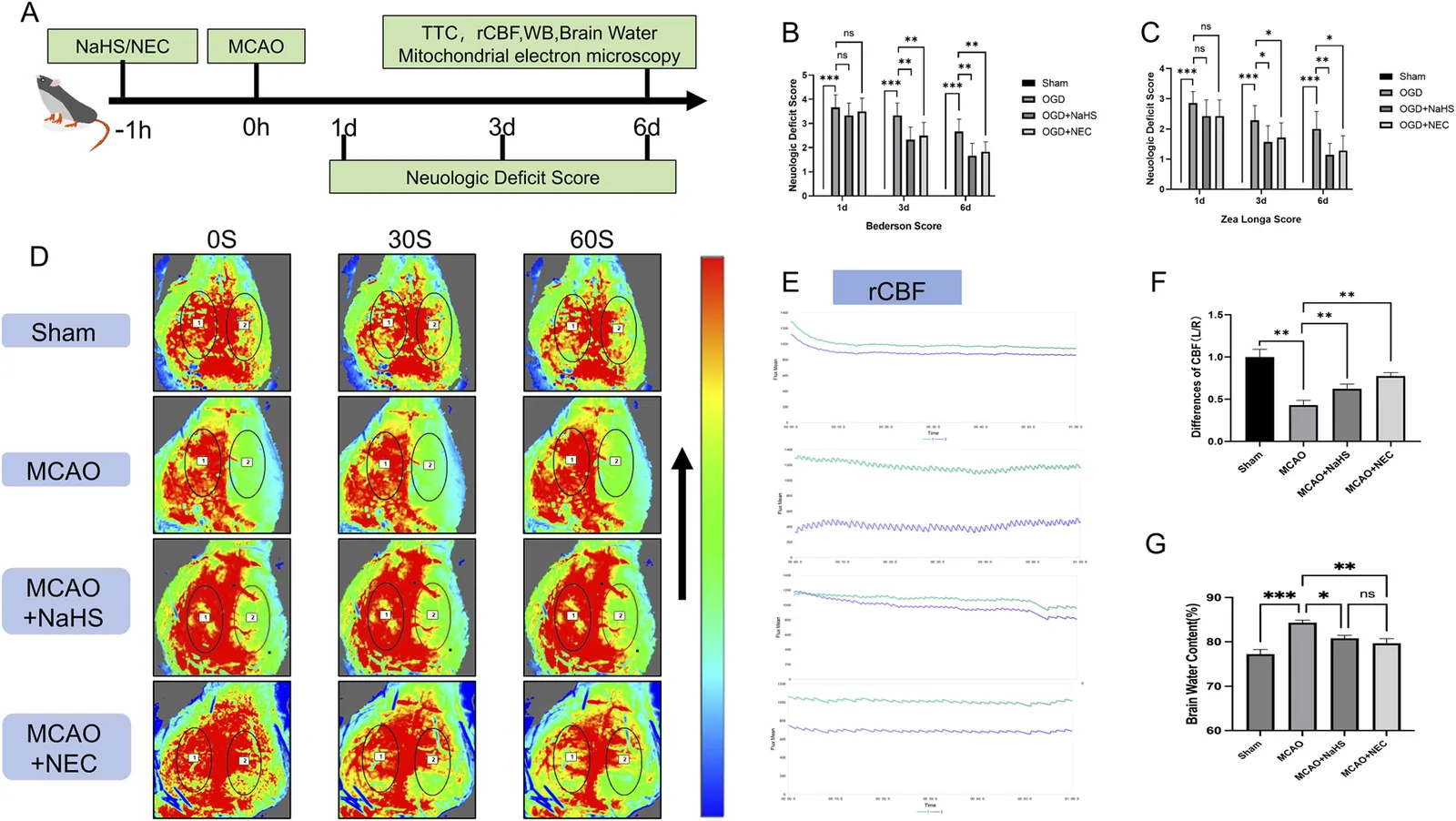
Exogenous H_2_S inhibited necrptosis induced by MCAO/R injury *in vivo*. **(A)**Morphological changes of Mouse brain tissue under TEM. The significant reduction by NaHS and Nec-1 markedly alleviated ultrastructural disruptions in neuronal nuclei, mitochondria, and other organelles caused by cerebral MCAO/R injury. The neuroprotective effectiveness of NaHS was similar to that of Nec-1, indicating that NaHS protected brain tissues from cerebral MCAO/R injury by suppressing necroptosis. **(B,C)**TTC staining was employed to evaluate the extent of cerebral infarction. In comparison to the model group, the NaHS group and the NEC group exhibited significantly reduced infarct sizes. **(D)**Western blot analysis demonstrated a significant increase in the expression of RIPK1 and RIPK3 in brain tissue following MCAO/R injury. Both NaHS and Nec-1 attenuated the upregulation of RIPK1 and RIPK3. **(E,F)**represent the relative expression levels of RIPK1 and RIPK3 respectively. Scale bar = 40 mm.

### 3.6 Exogenous H_2_S improved rCBF, cerebral edema, and infarct volume in mice following MCAO/R injury

Laser speckle contrast imaging (LSCI) was utilized to dynamically capture changes in regional Cerebral Blood Flow (rCBF) in both cerebral hemispheres at 0, 30 s, and 60 s (Figure 5D). In the Sham group, consistently high rCBF levels (mainly red signals) were symmetrically observed in both the ipsilateral (ROI 1) and contralateral (ROI 2) brain regions throughout the observation period, indicating no apparent perfusion abnormalities. Conversely, the untreated MCAO group displayed significant hypoperfusion in the ischemic ipsilateral hemisphere at all time points examined; the ipsilateral ROI exhibited distinct green/blue signals, confirming a substantial reduction in focal cerebral blood flow following artery occlusion.

Administration of NaHS or Nec-1 significantly alleviated the ischemic hypoperfusion phenotype in mice subjected to MCAO. In comparison to the vehicle-treated MCAO group, the MCAO + NaHS and MCAO + NEC groups exhibited markedly increased red/yellow perfusion signals within the ipsilateral infarct core region at 0, 30 s, and 60 s, indicating preserved microcirculatory perfusion. Quantitative temporal rCBF curves, derived from region-of-interest analysis, are illustrated in Figures 5E,F. Continuous perfusion tracing confirmed that baseline rCBF remained stable in Sham animals throughout the recording period. The MCAO group displayed persistently suppressed rCBF values, whereas NaHS or Nec-1 intervention significantly elevated sustained ipsilateral rCBF levels compared to untreated MCAO mice over the entire observation period. Collectively, real-time LSCI perfusion imaging demonstrated that exogenous NaHS supplementation effectively mitigated MCAO/R induced cerebral hypoperfusion and preserved regional cerebral blood flow in the ischemic brain territory.

We further quantified cerebral edema by measuring brain water content across all four experimental groups (Figure 5G). Brain water content was significantly elevated in the MCAO group compared to the Sham group (***P < 0.001), indicating severe ischemia-induced cerebral edema following middle cerebral artery occlusion. Treatment with NaHS or Nec-1 group significantly reduced brain water content relative to vehicle-treated MCAO animals (*P < 0.05), demonstrating that NaHS and Nec-1 intervention alleviated ischemic brain edema. No statistically significant difference in brain water content was found between the NaHS group and the NEC group (ns, not significant), suggesting comparable anti-edema efficacy between these two treatments. Additionally, the brain water content in both the NaHS and NEC groups remained markedly higher than that of the Sham group (*P < 0.05). Collectively, these measurements of brain water content corroborated the rCBF imaging findings, indicating that NaHS could ameliorate MCAO/R induced cerebral hypoperfusion and subsequent cerebral edema.

2,3,5-triphenyltetrazolium chloride (TTC) staining was employed to evaluate the extent of cerebral infarction. The infarct area in the model group was pronounced (Figure 6B), comprising 38.29% of the brain, which indicates successful establishment of the MCAO model. In comparison to the model group, the NaHS group (27.55% ± 3.01%) and the NEC group (23.82% ± 4.65%) exhibited significantly reduced infarct sizes (P < 0.01, Figure 6C). This finding further suggests the involvement of necroptosis among the various forms of programmed cell death (PCD) observed in the mouse MCAO model.

**FIGURE 6:**
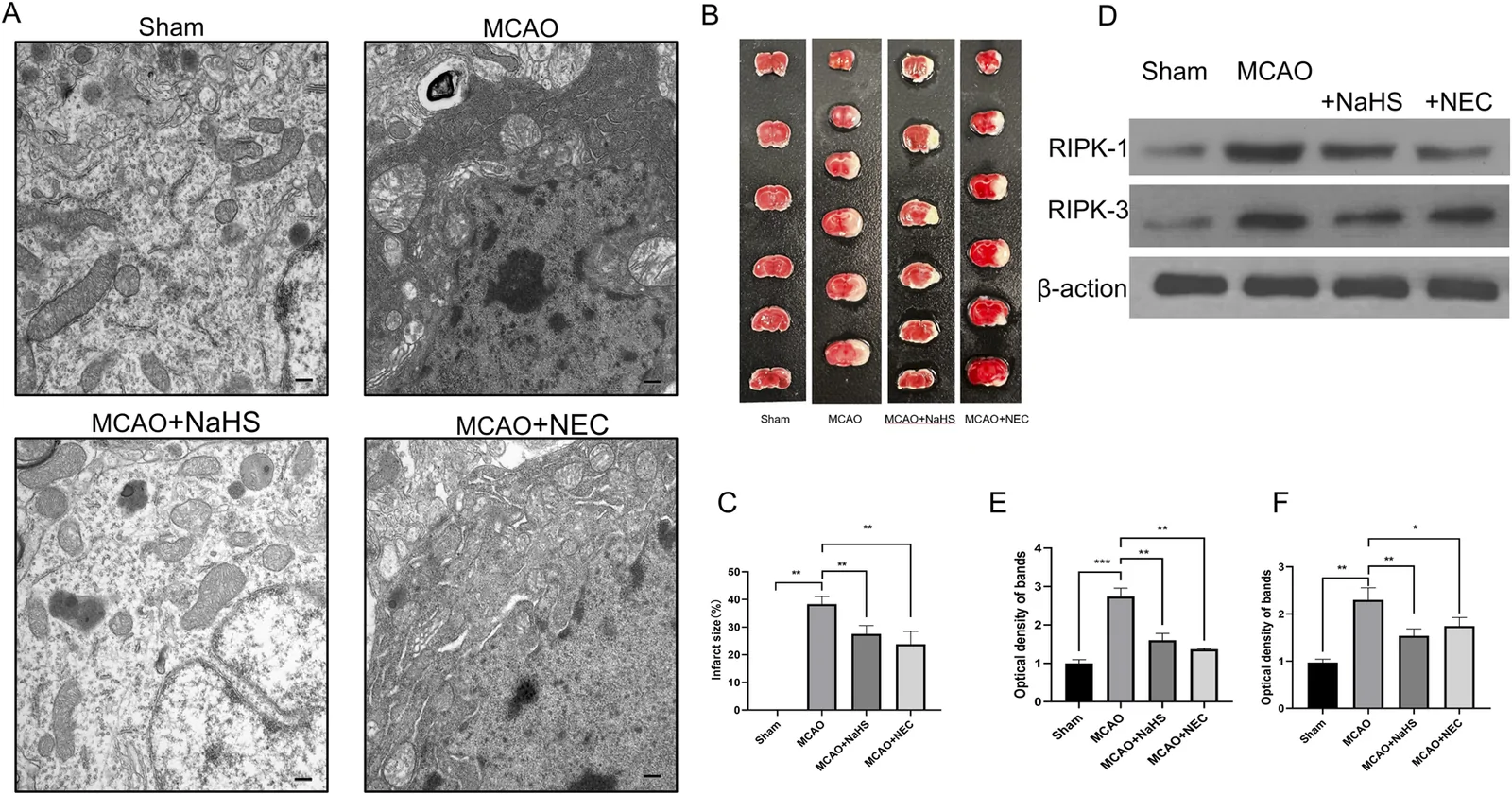
Exogenous H_2_S ameliorated neurological function, improved rCBF, cerebral edema, and infarct volume in mice after MCAO/R injury. **(A)**The procedure for the mouse experiment in this study. **(B,C)**The Bederson and Zea Longa scales were used to assess Neurological deficit scores, the Bederson scores and Zea Longa scales in the NaHS group and the NEC group were significantly lower than those in the MCAO group on the third and the sixth days after MCAO/R treatment. **(D)**LSCI was utilized to dynamically capture changes in rCBF in both cerebral hemispheres at 0 s, 30 s, and 60 s, the results showed that administration of NaHS or Nec-1 significantly alleviated the ischemic hypoperfusion phenotype in mice subjected to MCAO. **(E,F)**Quantitative temporal rCBF curves, derived from region-of-interest analysis. G:quantified cerebral edema by measuring brain water content, Treatment with NaHS or Nec-1 group significantly reduced brain water content relative to vehicle-treated MCAO animals demonstrating that NaHS and Nec-1 intervention alleviated ischemic brain edema.

### 3.7 Morphological changes of mouse brain tissue under TEM

Transmission electron microscopy (TEM) was utilized to examine the ultrastructural morphology of neurons in the brain tissues of mice from four experimental groups (Figure 6A). In the Sham group, neuronal nuclei displayed regular contours with evenly distributed chromatin, along with structurally intact organelles such as mitochondria and endoplasmic reticulum. Clear mitochondrial cristae devoid of swelling or vacuolation were evident. Conversely, the MCAO group exhibited severe ultrastructural damage in neurons, characterized by extensive chromatin condensation in the nucleus, pronounced mitochondrial swelling with disrupted cristae, disintegrated endoplasmic reticulum, and widespread cytoplasmic vacuolization, indicative of necrotic features. Remarkably, neuronal injuries affecting nuclei, mitochondria, and endoplasmic reticulum were significantly mitigated in both the MCAO + NaHS and MCAO + NEC groups, where the structural integrity of organelles was substantially restored, underscoring the notable neuroprotective effects of these interventions. These results collectively illustrated the substantial attenuation by NaHS of the cerebral MCAO/R injury induced ultrastructural disturbances in neuronal nuclei, mitochondria, and other organelles. Importantly, the neuroprotective efficacy of NaHS was comparable to that of Nec-1, a specific pharmacological inhibitor of necroptosis, suggesting that NaHS safeguarded brain tissues against cerebral MCAO/R injury by inhibiting necroptosis.

### 3.8 Exogenous H_2_S inhibited necrptosis induced by MCAO/R injury by suppressing the RIPK1-RIPK3 signaling pathway *in vivo*

Western blotting was utilized to quantify the expression of necroptosis-related proteins, specifically RIPK1 and RIPK3. In Figures 6D–F, it is evident that the MCAO group exhibited a significant upregulation in the expression of RIPK1 and RIPK3 compared to the sham operation group. Conversely, both the NaHS group and NEC group demonstrated a significant downregulation in the expression of RIPK1 and RIPK3 compared to the MCAO group. These findings suggested that exogenous sodium hydrosulfide and Nec-1 effectively inhibited necroptosis induced by OGD through the suppression of the RIPK1-RIPK3 signaling pathway.

## 4 Discussion

The study primarily focuses on investigating the CIRI mechanism as a key pathophysiological factor in acute cerebral ischemic diseases. Our findings support the hypothesis that the morphological and molecular changes associated with neuronal death in acute I/R injury correspond to necroptosis characteristics and implicate ROS in RIPK-dependent neuronal necroptosis following cerebral I/R injury. This underscores the crucial role of necroptosis in the development and advancement of cerebral I/R injury. Additionally, H_2_S can effectively alleviate I/R-induced changes by suppressing the overexpression of RIPK and ROS generation, thereby safeguarding the brain against I/R-induced damage and functional impairments. This research offers novel insights into the pathological mechanisms of neuronal damage post I/R injury and highlights the potential of H_2_S in inhibiting necroptosis in cerebral I/R injury, providing valuable experimental support for the clinical use of H_2_S in ischemic stroke treatment.

Recent studies have identified CIRI mechanisms such as neuroinflammation (Wu et al., 2018), oxygen free radical damage (Orellana-Urzua et al., 2020), and calcium overload (Han et al., 2022). These mechanisms can ultimately result in mitochondrial dysfunction, disruption of the blood-brain barrier, and neuronal necrosis (He et al., 2020; Jurcau and Ardelean, 2021), contributing to cerebral I/R injury characterized by apoptosis, necrosis, necroptosis, and autophagy. The specific type of cell death is associated with ischemia duration, oxygen levels, and residual blood flow. Moderate cerebral I/R injury may induce cell autophagy and activate protective mechanisms for cell survival, whereas severe injury predominantly triggers apoptotic and necrotic pathways (Zhang et al., 2022). Previous studies have reported the presence of necroptosis in cerebral I/R (Vieira et al., 2014), supporting the reliability of our experimental results. Nonetheless, discrepancies in findings across studies may arise from variations in cell types, time points of measurement, or research methodologies (Wu et al., 2012). Further investigation is needed to elucidate the precise mechanism of necroptosis. This study employed experimental conditions of 2–12 h of OGD followed by 24 h of reoxygenation. Within this timeframe, the phosphorylation of key molecules in the necroptosis pathway, RIPKs, and MLKL, was significantly activated, serving as the primary mechanism driving neuronal death. Importantly, our study on the morphological changes of neuronal death in acute I/R injury reveals significant characteristics, such as extensive chromatin condensation in the nucleus, pronounced mitochondrial swelling with disrupted cristae, fragmented endoplasmic reticulum, and widespread cytoplasmic vacuolization Moreover, we observed a substantial increase in the expression levels of RIPK and MLKL, underscoring the crucial role of necroptosis in the initiation and progression of cerebral I/R injury.

Necroptosis appears to be the primary form of programmed cell death at the onset of cerebral I/R injury (Liu et al., 2025). In the model of MCAO, it was observed that RIPK1 activation sequentially induced necroptosis followed by apoptosis in a time-specific manner due to TAK1 degradation (Naito et al., 2020). Necroptosis is triggered by the activation of RIPK1/3 and MLKL. RIPK1 is crucial in regulating necroptosis in cerebral I/R injury (Wang et al., 2024). Consequently, modulating the kinase activity of RIPK1 has emerged as a critical approach to mitigate cerebral I/R injury.

For most researchers, the primary focus is on discovering effective methods to prevent ischemic brain injury. The growing comprehension of the properties of H_2_S has sparked considerable interest among scholars due to its notable role in the pathogenesis of CIRI. Previous research has demonstrated the substantial neuroprotective effects of H_2_S in animal models of Alzheimer’s disease, Parkinson’s disease, cerebral ischemia, and various other neurological conditions (Hu et al., 2010; Nagpure and Bian, 2015; Tao et al., 2019). Despite the intricate and varied mechanisms at play, these preclinical studies have been conducted to unveil protective strategies aimed at enhancing the management of brain ischemia-reperfusion injury.

During brain I/R injury, localized accumulation of ROS in ischaemic brain tissue can lead to dysfunction of ion transport proteins in cell membranes and increased vascular permeability, resulting in adverse outcomes such as mitochondrial dysfunction, brain edema, and cell death, ultimately causing secondary damage (Huang et al., 2023). Previous research has shown that necroptosis generates a substantial amount of ROS, and excessive ROS can further stimulate RIP1 expression, worsening necroptosis (Huang et al., 2013). Our study aims to elucidate the protective effects and potential mechanisms of H_2_S in CIRI through the lens of necroptosis.

This report represents the initial investigation providing evidence that H_2_S may reverse cell necroptotic programmed cell death, highlighting the multifaceted protective roles of hydrogen sulfide in brain cell preservation. Our findings illustrate that H_2_S exhibited protective effects against neuronal damage induced by OGD/R through the specific inhibition of RIPK1-mediated necroptosis, as well as reducing the generation of ROS. This interference disrupted subsequent signaling cascades by decreasing RIPK3 phosphorylation, inhibiting MLKL activation, and impeding necrosome complex assembly, ultimately halting necroptotic cell demise. The direct interaction between H_2_S and downstream molecules of RIPK-1, including RIPK3 or MLKL, was not thoroughly explored in this study. Moreover, hydrogen sulfide may potentially exert direct inhibitory effects on Ca^2+^ influx and facilitate the restoration of mitochondrial oxidative stress damage; however, a more comprehensive exploration of these intricate regulatory pathways is warranted.

Our study demonstrates that I/R injury enhances the expression of RIPS and the production of ROS both *in vitro* and *in vivo*, thereby promoting necroptosis. In contrast, H_2_S mitigates these effects, preventing necroptosis and offering protection against I/R injury. Consequently, we established that H_2_S is critical for necroptosis by modulating the kinase activity of RIPK1 and inhibiting ROS generation in cerebral I/R injury. These findings provide direct *in vitro* and *in vivo* evidence that H_2_S plays a significant role in inhibiting RIPK1-dependent necroptosis in the pathogenesis of I/R injury in the rodent brain.Despite the promising neuroprotective outcomes observed in this study, several inherent limitations should be acknowledged. First, all phenotypic and mechanistic validations were primarily performed using *in vitro* cell models and *in vivo* animal paradigms, without verification in clinical tissue samples, which compromises the translational credibility of the present therapeutic findings. Second, the *in vivo* data were acquired from preliminary exploratory experiments with a relatively small sample size (n = 3 per group), which may undermine statistical power; follow-up studies with enlarged animal cohorts are therefore required to consolidate our observations. Third, the lack of gene knockout validation limits the robustness of the causal mechanistic evidence presented in this work. Fourth, the current study adopted a preconditioning strategy to explore the priming effects of H_2_S on endogenous protective signaling prior to ischemic injury. Notably, preconditioning regimens cannot fully recapitulate the pathological characteristics of clinical ischemic stroke. Hence, subsequent studies will focus on post-ischemic H_2_S administration to better simulate clinical scenarios and improve the translational relevance of our findings.

Beyond the above experimental limitations, the clinical transformation of H_2_S-based therapy still faces multiple inherent challenges. Although H_2_S exerts potent neuroprotective effects against OGD/R-induced neuronal damage in preclinical models, direct inhalation of gaseous H_2_S is constrained by inherent safety hazards and poor concentration controllability, while H_2_S-releasing donors serve as a more feasible alternative. In addition, heterogeneous therapeutic responses among individual patients may arise from variations in genetic background, metabolic status, and comorbidities. Critically, H_2_S possesses a narrow therapeutic window and potential off-target adverse effects, which cannot be overlooked in clinical practice. Furthermore, the efficacy of H_2_S treatment against cerebral ischemia induced by diverse etiologies, including trauma, biochemical insults, and drug-related injury, remains to be systematically validated. Collectively, the findings of the present study are preliminary, and rigorous *in vivo* validation and clinical trials are indispensable before widespread clinical application. Specifically, we will incorporate clinical tissue samples and establish standardized, clinically relevant models to further confirm the efficacy of H_2_S in alleviating I/R injury-triggered cellular necroptosis. Moreover, we will systematically explore the regulatory effects of H_2_S on downstream signaling molecules and transcription factors associated with programmed cell death, aiming to refine and complete the molecular regulatory network underlying H_2_S-mediated neuroprotection.

## 5 Conclusion

The findings revealed that I/R injury significantly damages brain cells by inducing RIPK1 mediated necroptosis. H_2_S or Nec-1 can regulate the expression of RIPK1, thereby modulating necroptosis following cerebral I/R injury. We provide evidence for the first time that H_2_S directly targets RIPK1 and ROS, thereby attenuating RIPK1 mediated necroptosis. Given the critical role of H_2_S in cerebral I/R injury, it represents a promising therapeutic agent for stroke treatment.

## Data Availability

The original contributions presented in the study are included in the article/supplementary material, further inquiries can be directed to the corresponding authors.
